# Conditional survival analysis and real-time prognosis prediction for cervical cancer patients below the age of 65 years

**DOI:** 10.3389/fonc.2022.1049531

**Published:** 2023-01-09

**Authors:** Xiangdi Meng, Yingxiao Jiang, Xiaolong Chang, Yan Zhang, Yinghua Guo

**Affiliations:** ^1^ Department of Radiation Oncology, Weifang People’s Hospital, Weifang, Shandong, China; ^2^ School of Clinical Medicine, Weifang Medical University, Weifang, China

**Keywords:** cervical cancer, conditional survival, cancer-specific survival, nomogram, SEER

## Abstract

**Background:**

Survival prediction for cervical cancer is usually based on its stage at diagnosis or a multivariate nomogram. However, few studies cared whether long-term survival improved after they survived for several years. Meanwhile, traditional survival analysis could not calculate this dynamic outcome. We aimed to assess the improvement of survival over time using conditional survival (CS) analysis and developed a novel conditional survival nomogram (CS-nomogram) to provide individualized and real-time prognostic information.

**Methods:**

Cervical cancer patients were collected from the Surveillance, Epidemiology, and End Results (SEER) database. The Kaplan–Meier method estimated cancer-specific survival (CSS) and calculated the conditional CSS (C-CSS) at year y+x after giving x years of survival based on the formula C-CSS(y|x) =CSS(y+x)/CSS(x). y indicated the number of years of further survival under the condition that the patient was determined to have survived for x years. The study identified predictors by the least absolute shrinkage and selection operator (LASSO) regression and used multivariate Cox regression to demonstrate these predictors’ effect on CSS and to develop a nomogram. Finally, the CSS possibilities predicted by the nomogram were brought into the C-CSS formula to create the CS-nomogram.

**Results:**

A total of 18,511 patients aged <65 years with cervical cancer from 2004 to 2019 were included in this study. CS analysis revealed that the 15-year CSS increased year by year from the initial 72.6% to 77.8%, 84.5%, 88.8%, 91.5%, 93.5%, 94.8%, 95.7%, 96.4%, 97.3%, 98.0%, 98.5%, 99.1%, and 99.4% (after surviving for 1-13 years, respectively), and found that when survival exceeded 5-6 years, the risk of death from cervical cancer would be less than 5% in 10-15 years. The CS-nomogram constructed using tumor size, lymph node status, distant metastasis status, and histological grade showed strong predictive performance with a concordance index (C-index) of 0.805 and a stable area under the curve (AUC) between 0.795 and 0.816 over 15 years.

**Conclusions:**

CS analysis in this study revealed the gradual improvement of CSS over time in long-term survived cervical cancer patients. We applied CS to the nomogram and developed a CS-nomogram successfully predicting individualized and real-time prognosis.

## Introduction

Cervical cancer is the fourth most common cause of cancer death among women worldwide (1, 2), which best occurs between the ages of 35 and 55, and about 20% of women are over 65 years old, rarely under 20 years old (1, 3). With advances in screening, genital hygiene, vaccination, and treatment, the mortality rate from cervical cancer has declined significantly, with deaths decreasing from 8.2% of all cancers in 2008 to 7.5% in 2018 (1, 3). Long-term survivors may now be more concerned about receiving accurate data on survival assessment. However, traditional survival analyses, estimated from the time of diagnosis, did not provide updated survival outcomes for those patients who survived for several years. Many studies have revealed significant improvements in long-term survival over time (4, 5), i.e., the 10-year survival probability predicted at the time of diagnosis differed from that predicted after patients survived for eight years. Therefore, traditional survival metrics may not provide such dynamic and real-time prognostic estimates.

Conditional survival (CS) is a promising survival metric that calculates survival over time and is defined as the probability that a patient who has survived for x years after diagnosis of cervical cancer will survive for another y years (4, 5). As we know, this metric has been applied in pancreatic adenocarcinoma (6, 7), gastric cancer (8), esophageal cancer (9), liver cancer (10), lung cancer (11), nasopharyngeal carcinoma (12), colorectal cancer (13), Hodgkin lymphoma (14), and melanoma (15), and dynamic improvements in prognosis over time have been observed. This favorable survival characteristics can provide significant support for cancer management, such as psychological support for patients, adjustment of follow-up frequency, optimal risk stratification, and guidance for adjuvant treatment decisions, which have important clinical value for patients and clinicians.

Although the traditional nomogram can individualize prognosis prediction (16, 17), it still cannot reveal the change in survival over time as CS does. Meanwhile, the CS model is imperfect, as it does not consider individualized prognostic factors. Excitingly, using CS in a nomogram may hold promise for individualized, real-time and dynamic prognostic prediction. Although previous studies calculated CS or developed nomograms for patients with cervical cancer (18–20), no combination of the two methods was reported. A novel conditional survival nomogram (CS-nomogram) could compensate for the shortcomings of CS and nomogram.

The aim of this study was to estimate conditional cancer-specific survival (C-CSS) for cervical cancer patients and develop the first CS-nomogram for providing accurate and real-time prognostic information for long-term survivors.

## Material and methods

### Data sources, patient selection and variables

This study used data from The Surveillance, Epidemiology, and End Results (SEER) database (17 Registries, updated in 2021), which had sufficient data for CS analysis. Before using the SEER database, we gained access to it (username: 11578-Nov2021), so this study did not require the consent of the institutional ethics committee.

Using the third edition of the International Classification of Diseases for Oncology (ICD-O-3), we identified the code for the cervix as C53 and screened 67,109 patients in the SEER database. The following exclusion criteria were used: (1) disease not diagnosed between 2004 and 2019; (2) the primary tumor was only cervical cancer; (3) age <18 or >65 years old; (4) the necessary variables were unknown; (5) not confirmed by positive histology; (6) the follow-up period was 0 months. In addition, age at diagnosis, race, marital status, histological grade, pathological type, tumor size, lymph node metastasis, distant metastasis, surgery, chemotherapy, and radiation therapy were included in this study. In addition, previous studies suggested that the interval between diagnosis and treatment may affect patient survival (21), so we excluded patients with an unknown time from diagnosis to treatment. Ultimately, these screened patients were divided into training and validation groups according to the ratio of 7:3.

### Statistical analysis

Categorical variables in this study reported percentages and numbers, and continuous variables reported means and standard deviations (SD) or median and interquartile range (IQR), depending on whether they met normality. The clinical endpoint of the study was CSS, defined as the time from the patient’s diagnosis of cervical cancer to death due to cervical cancer and estimated by the Kaplan-Meier method.

Conditional CSS (C-CSS) was calculated according to the formula C-CSS(y|x) = CSS(y+x)/CSS(x). In this formula, C-CSS(y|x) was the probability of surviving further y years, given that a patient has already survived x years after a cervical cancer diagnosis. Moreover, CSS(x) and CSS(y+x) were the CSS of the patient for x- and (x+y)-years estimated by the Kaplan-Meier method. For example, if the patient has survived 8 years after the diagnosis of cervical cancer and wants to know the probability of surviving another 2 years, the result was calculated as C-CSS(2|8) =CSS(2 + 8)/CSS (8) =10-years CSS/8-years CSS.

This study used the least absolute shrinkage and selection operator (LASSO) regression plus 10-fold cross-validation for confirming predictors to avoid overfitting. Then, one standard error of the minimum mean squared error (MSE) was used as a screening criterion. The screened predictors revealed their effect on cervical cancer survival by multivariate Cox regression and were used to develop a nomogram. The current study added the CS formula to the nomogram so that this model could consider individualized predictors and dynamic survival time for patients with cervical cancer. The CS-nomogram quantified the predictors as points, and inputting the individualized variables of the patient could result in a total point, which corresponded to the patient’s survival rate. Most importantly, this survival data was continuously updated as survival time increased. For example, a patient with a total point of 180 corresponds to a 15-year CSS of 35% at diagnosis and an OS adjustment of 75% after 5 years of follow-up (estimated using the 15-year C-CSS (10|5) probability axis).

Subsequently, the model was evaluated and validated in the training and validation groups. The concordance index (C-index) assessed the discrimination, and the closer its value was to 1.0, the better the model discrimination was. Model stability was estimated by time-dependent receiver operating characteristic (ROC) and time-dependent C-index. If the fluctuations of the area under the curve (AUC) at 5-, 10-, and 15 years or annual C-index were fluctuating little, the model stability did not decrease with survival time. The calibration plots assessed the accuracy of the model. The closer the calibration curves were to the ideal 45° line, the closer the probability predicted by the nomogram was to the actual. The clinical usefulness was tested using decision curve analysis (DCA), which assessed the net benefit that may be derived from using the nomogram. The statistical analysis of this study was done by R (version 4.1.0). P values less than 0.05 were considered statistically significant in the two-tailed test.

## Results

### Clinicopathological characteristics

**Figure 1:**
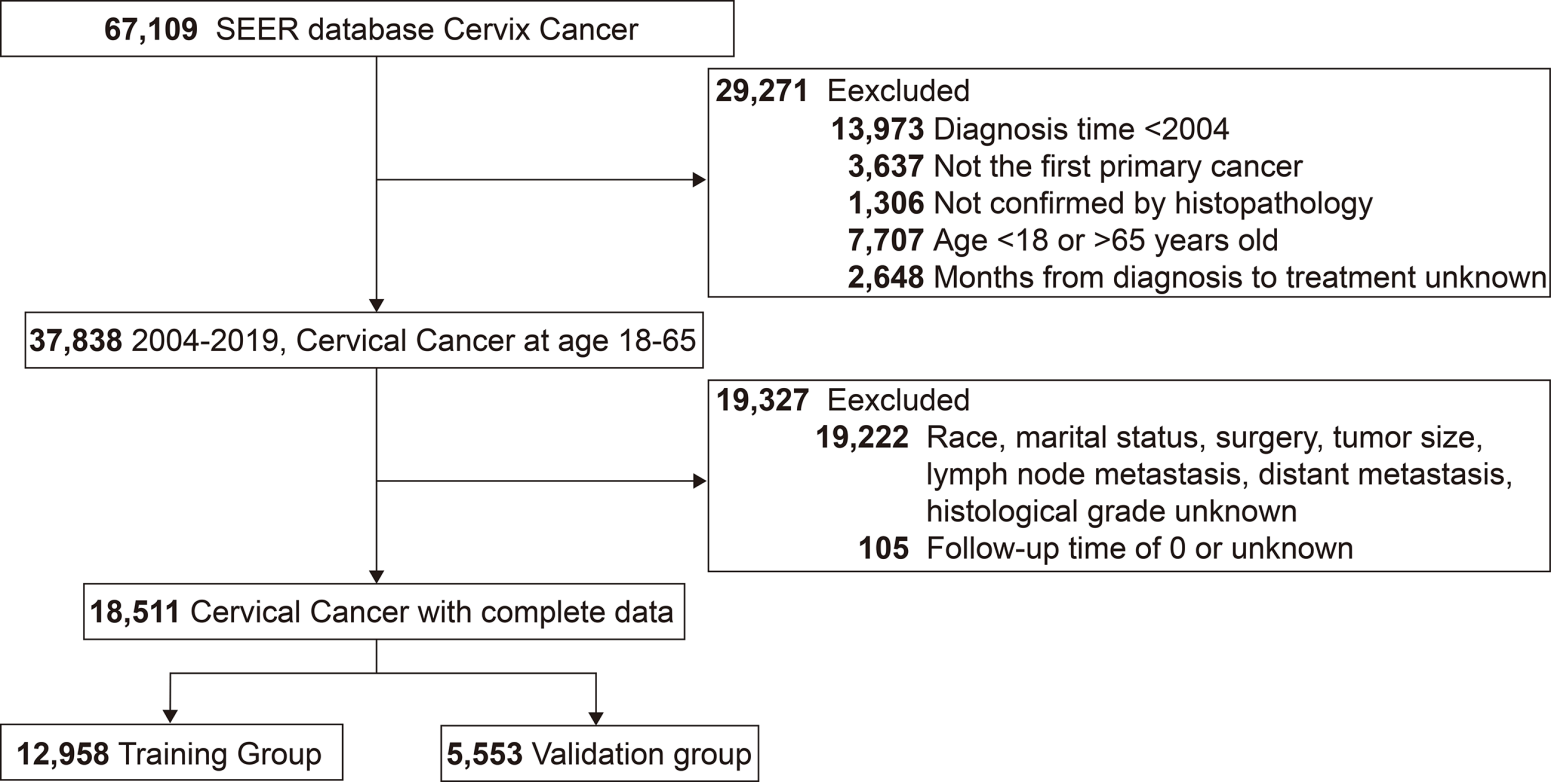
Flow chart for screening patients with cervical cancer. SEER, the Surveillance, Epidemiology, and End Results.

A total of 18,511 patients pathologically diagnosed with cervical cancer between 2004 and 2019 were included in this study, with 12,958 entering the training group and 5,553 in the validation group. The detailed screening process was shown in the flow chart (**Figure 1**). The mean age of the patients was 44.7 years (SD=10.4 years), with a median follow-up of 56 months (IQR: 23-112 months) and a predominantly white population (77.7%, 14380/18511). The pathological type was predominantly squamous carcinoma in 64.1% (11,861/18511), followed by adenocarcinoma in 29.1% (5,390/18511). At diagnosis, 60% (11104/18511) of patients had tumors <40 mm in size, 75.2% (13922/18511) had negative lymph nodes, and only 1460 (7.9%) patients had distant metastases. See **Table 1** for details.

**Table 1:** Patient clinicopathologic characteristics in cervical cancer.

Characteristics	Whole cohort	Training group	Validation group	P value
	n=18,511 (%)	n=12,958 (%)	n=5,553 (%)	
Age at diagnosis, years				
Mean (SD)	44.7 (10.4)	44.7 (10.4)	44.8 (10.4)	0.341
18~25	326 (1.8)	227 (1.8)	99 (1.8)	0.943
26~45	9721 (52.5)	6815 (52.6)	2906 (52.3)	
46~65	8464 (45.7)	5916 (45.7)	2548 (45.9)	
Race				0.108
Black	2037 (11.0)	1423 (11.0)	614 (11.1)	
White	14380 (77.7)	10110 (78.0)	4270 (76.9)	
Other	2094 (11.3)	1425 (11.0)	669 (12.0)	
Marital status				0.711
Married	9134 (49.3)	6406 (49.4)	2728 (49.1)	
Unmarried	9377 (50.7)	6552 (50.6)	2825 (50.9)	
Pathological type				0.969
Squamous carcinoma	11861 (64.1)	8317 (64.2)	3544 (63.8)	
Adenocarcinoma	5390 (29.1)	3764 (29.0)	1626 (29.3)	
Adenosquamous carcinoma	834 (4.5)	580 (4.5)	254 (4.6)	
Other	426 (2.3)	297 (2.3)	129 (2.3)	
Site				0.436
Cervix uteri	13481 (72.8)	9422 (72.7)	4059 (73.1)	
Endocervix	4205 (22.7)	2939 (22.7)	1266 (22.8)	
Exocervix	444 (2.4)	317 (2.4)	127 (2.3)	
Other	381 (2.1)	280 (2.2)	101 (1.8)	
Tumor size, mm				0.260
≤20	6522 (35.2)	4517 (34.9)	2005 (36.1)	
20~40	4582 (24.8)	3231 (24.9)	1351 (24.3)	
>40	7407 (40.0)	5210 (40.2)	2197 (39.6)	
Lymph node metastasis				0.248
No	13922 (75.2)	9714 (75.0)	4208 (75.8)	
Yes	4589 (24.8)	3244 (25.0)	1345 (24.2)	
Distant metastasis				0.742
No	17051 (92.1)	11942 (92.2)	5109 (92.0)	
Yes	1460 (7.9)	1016 (7.8)	444 (8.0)	
Histological grade				0.301
I	2964 (16.0)	2051 (15.8)	913 (16.4)	
II	8123 (43.9)	5666 (43.7)	2457 (44.2)	
III-IV	7424 (40.1)	5241 (40.4)	2183 (39.3)	
Surgery				0.529
No	5281 (28.5)	3701 (28.6)	1580 (28.5)	
Local tumor excision	1850 (10.0)	1274 (9.8)	576 (10.4)	
Total hysterectomy	11380 (61.5)	7983 (61.6)	3397 (61.2)	
Radiotherapy				0.582
No	8072 (43.6)	5633 (43.5)	2439 (43.9)	
Yes	10439 (56.4)	7325 (56.5)	3114 (56.1)	
Chemotherapy				0.733
No	9340 (50.5)	6527 (50.4)	2813 (50.7)	
Yes	9171 (49.5)	6431 (49.6)	2740 (49.3)	

### Conditional cancer-specific survival analysis

3939 (21.3%) patients in this study died because of cervical cancer. Kaplan-Meier curve showed that patients had a CSS of 77.7% (95% confidence interval (CI): 77.0%-78.3%), 74.1% (95% CI: 73.4%-74.8%), and 72.6% (95% CI: 71.8-73.5) at 5, 10 and 15 years, respectively.

**Figure 2:**
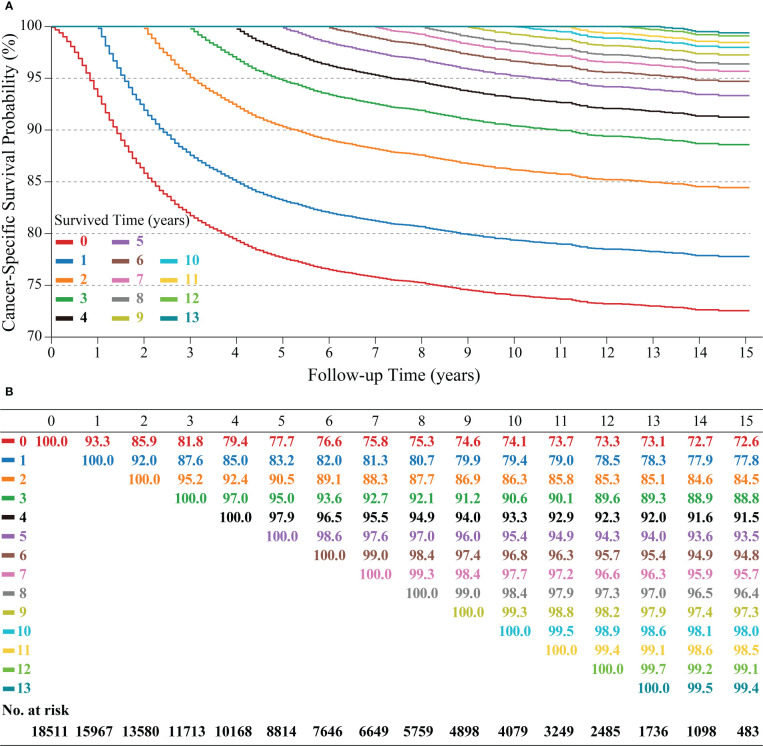
Kaplan–Meier method for estimating conditional cancer-specific survival (C-CSS) at 15 years after surviving 0~13 years in cervical cancer patients. Conditional survival curves **(A)** and their updated survival data adjusted for survived time **(B)**.

C-CSS was assessed in long-term surviving cervical cancer patients using CS analysis (**Figure 2**). Each curve recorded the change in the patient CSS for each additional year of survival (**Figure 2A**). In addition, the table recorded CSS at each follow-up time point, with each row representing CSS after patients who survived x-years and each column representing the CSS under different follow-up times (**Figure 2B**). C-CSS analysis showed that patients’ survival would gradually improve with each additional year of survival. For example, the 15-year CSS of patients increased year by year from the initial 72.6% to 77.8%, 84.5%, 88.8%, 91.5%, 93.5%, 94.8%, 95.7%, 96.4%, 97.3%, 98.0%, 98.5%, 99.1%, and 99.4% (corresponding to 1-13 years of follow-up, respectively). Meanwhile, we observed the most considerable interval of CS curves within 3 years after diagnosis, indicating the most significant improvement in survival. In addition, the CS curves became denser when survival was more than 5-6 years, conveying a lower real-time mortality rate for survivors at this time (**Figure 2A**).

### Development and validation of the CS-nomogram

**Figure 3:**
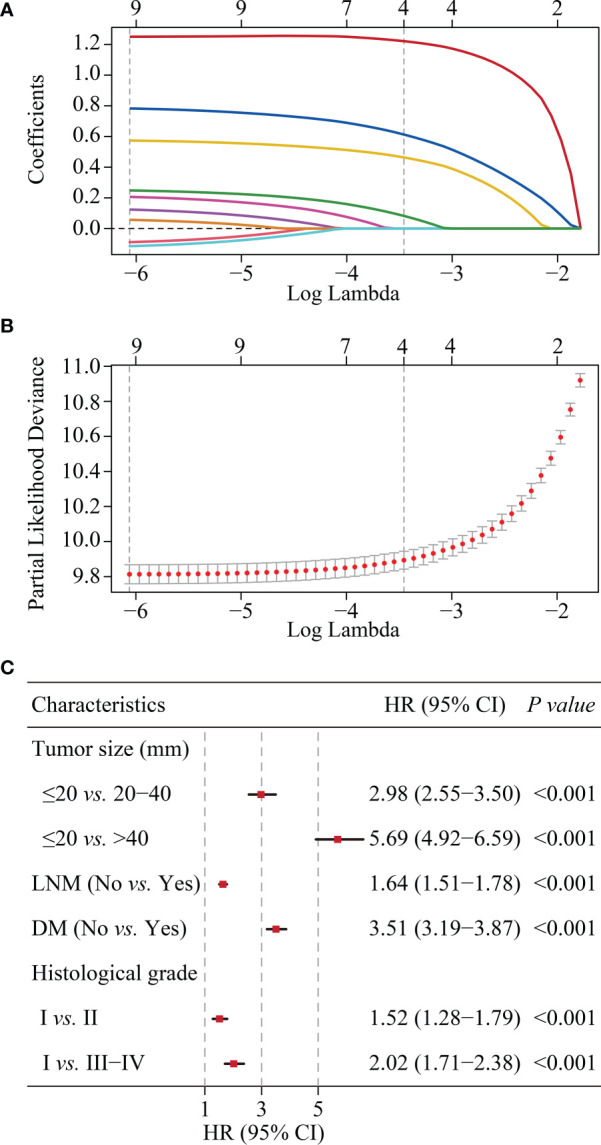
Predictor screening. **(A)** and **(B)**. The least absolute shrinkage and selection operator (LASSO) regression and 10-fold cross-validation for screening predictors. **(C)**. Multivariate Cox regression forest plot showing the effect of predictors on cancer-specific survival (CSS) of cervical cancer. HR, hazard ratio; CI, confidence interval; LNM, lymph node metastasis; DM, distant metastasis.

**Figure 4:**
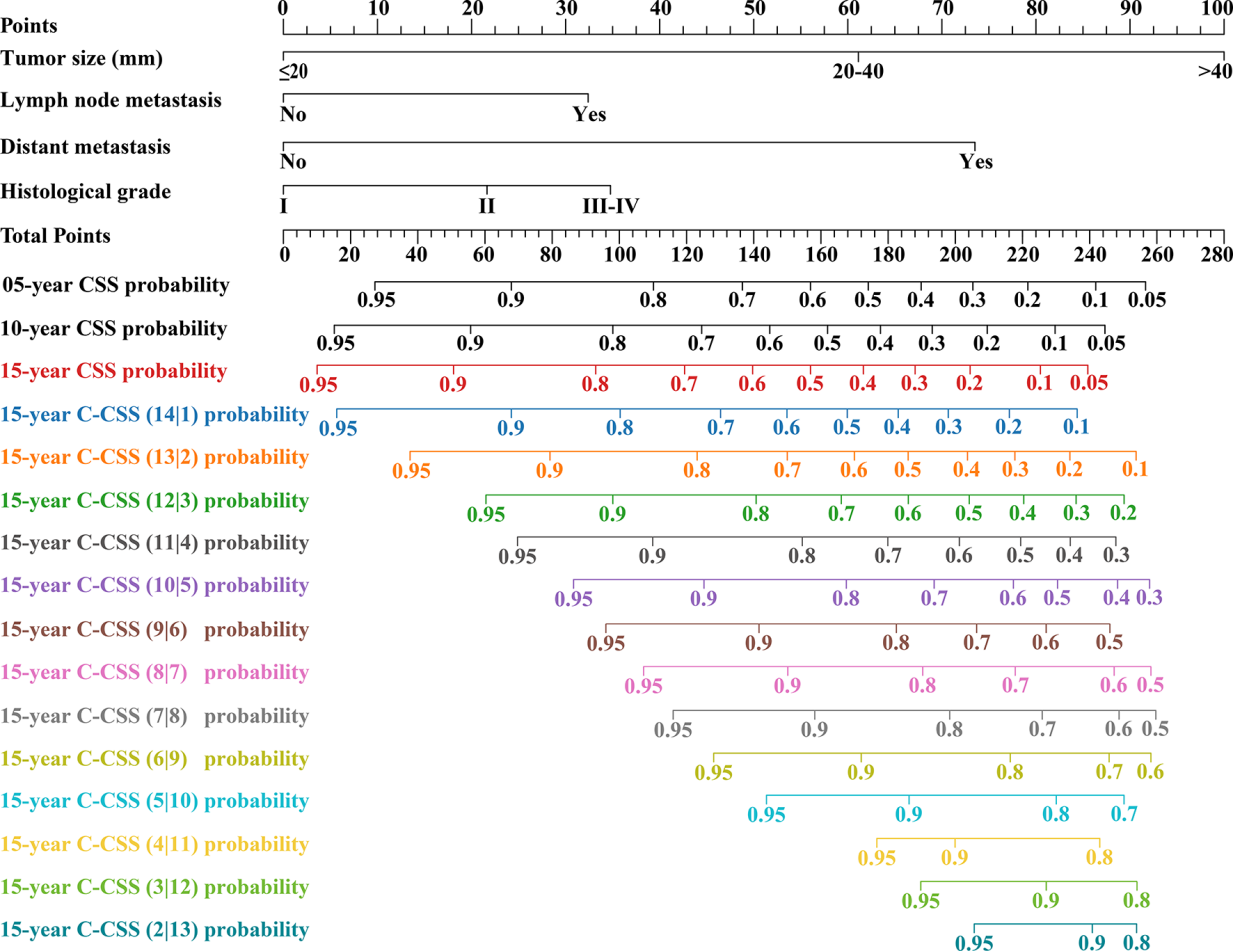
Conditional survival nomogram (CS-nomogram) for predicting 5-, 10- and 15-year cancer-specific survival (CSS) and 15-year conditional CSS (C-CSS) for cervical cancer.

According to LASSO regression, four clinicopathological factors were selected as predictors: tumor size, lymph node status, distant metastasis, and histological grade (**Figures 3A, B**). Multivariate Cox regression forest plot showed that all these predictors significantly affected CSS of cervical cancer and were used to develop the CS-nomogram (P<0.0001, **Figure 3C**). Unlike traditional prediction models, the CS-nomogram in this study took into account conditional survival, and patients were able to obtain not only 5-, 10- and 15-year CSS after inputting individualized clinicopathological factors but 15-year C-CSS based on the number of years they have survived since diagnosis (**Figure 4**).

**Figure 5:**
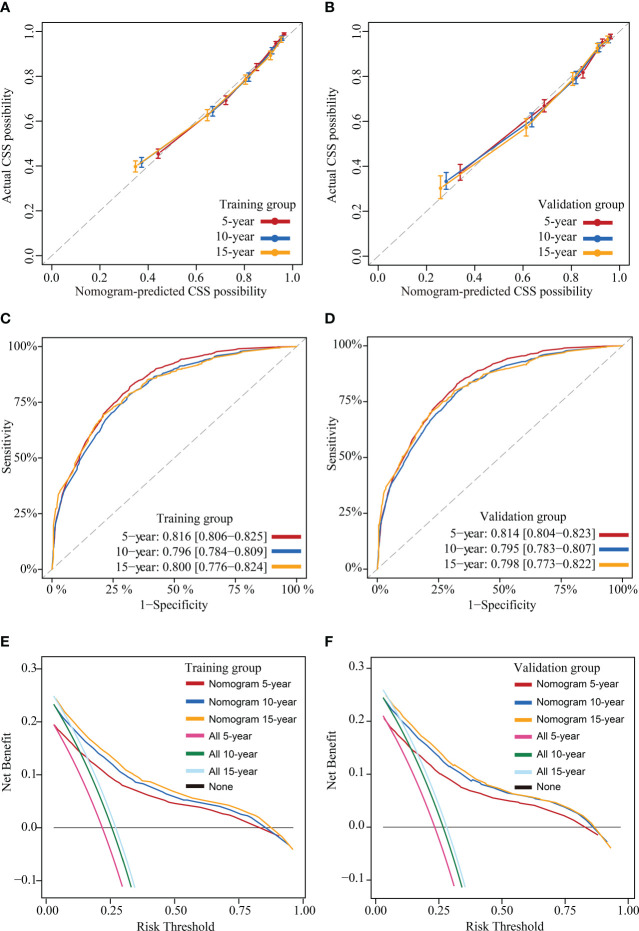
Model evaluation and validation. Calibration plots **(A)** and **(B)**, Time-dependent receiver operating characteristic (ROC) curves **(C)** and **(D)** and decision curve analysis (DCA) curves **(E)** and **(F)** for assessing the accuracy, discrimination and clinical usefulness of the conditional survival nomogram (CS-nomogram) at 5-, 10- and 15-years, respectively.

This study assessed the model’s performance in terms of discrimination, accuracy, temporal stability, and usefulness. The C-indexes measured in the training and validation groups were 0.805 (95% CI: 0.797-0.813) and 0.804 (95% CI: 0.796-0.812), respectively, and the calibration curves for these two groups at 5, 10 and 15 years showed strong agreement between the prediction of CS-nomogram and the actual (**Figures 5A, B**), and the time-dependent ROC curves and the time-dependent C-indexes indicated that the model was very stable in predicting survival over 15 years (**Figures 5C, D** and **Supplementary Figure 1**). In addition, the DCA curves showed that there was always a good net benefit when medical interventions were triggered using the CS-nomogram (**Figures 5E, F**).

## Discussions

CS analysis in this study indicated that CSS improved gradually with the years the cervical cancer patient had survived. To obtain individualized and real-time survival assessments, we developed the first CS-nomogram for long-term survivors. After rigorous evaluation and validation, this model with strong predictive performance was expected to provide patients and clinicians with a valuable reference for follow-up and treatment strategies.

Globally, although cervical cancer remains one of the most common cancers among women (22), its mortality rate has improved thanks to effective screening and treatment strategies (23). However, the interpretation of these improvements remains challenging. Because traditional survival estimates use only the patient’s initial diagnosis as a landmark and do not consider how long the patient has been followed up. Therefore, this survival prediction does not change either at the time of diagnosis or after the patient has survived for several years. This outcome is unfortunate for those who survived because they did not see any improvement in survival. CS analysis could give patients more hope. For example, a patient had a 10-year survival of 74.1% at initial diagnosis. However, after she adhered to treatment and follow-up and survived safely for 5 years, the CS analysis could tell her, “Your 10-year survival rate has increased by 21.3%”. Meanwhile, previous literature revealed that when conditional relative survival exceeded 95%, patient survival was almost similar to that of the general population of the same age structure (24–26). For long-term surviving cervical cancer patients in this study, when they survived beyond 5 years, their risk of death from cervical cancer was <5% in 10 years, and if they survived beyond 6 years, this time was 15 years. These favorable findings would help relieve patients’ anxiety, increase their confidence in fighting cancer, and improve their quality of life. In addition, mastering this dynamic survival pattern could help establish cost-effective surveillance strategies for cervical cancer in terms of duration and intensity of follow-up.

The second strength of our study was the consideration of individualized real-time survival prediction. CS estimated real-time survival outcomes for patients but failed to account for individualization. For example, there was a significant difference in the prognosis of early- and late-stage cervical cancer. Therefore, to address this deficiency, we used the CS-nomogram, which took into account the individualized clinicopathological factors of the patient. This study used tumor size, lymph node metastasis status, distant metastasis status and histological grade as predictors, which have been shown to correlate with prognosis and determine treatment strategy significantly. Meanwhile, since the stage of cervical cancer represents the treatment choice, the study did not consider using treatment factors as predictors to avoid multicollinearity. Tumor size >4 cm was a well-known risk factor, which was included in the FIGO staging system as stage IB2 (edition 2009) and IB3 (edition 2018) (22). In addition, recent studies, especially with the increasing availability of fertility-preserving surgery, suggested that tumor size >2 cm was a risk factor (22, 27–29), consistent with our Cox regression results. Lymph nodes and distant metastases meant that the disease was advanced, and the prognosis was poor. Notably, we did not use the FIGO or TNM staging systems in the current study because patients with the same staging may have different numbers of prognostic factors, which was not conducive to accurate prognostic prediction. In addition, histological grading also influenced patient survival as a poor prognostic factor in the studies of Bhatla et al., Liu et al. and Ni et al. (22, 30, 31). Ultimately, after confirming the availability of these four predictors, we developed the nomogram and incorporated CS into it to provide the first individualized real-time prognostic tool for cervical cancer patients. The advantage of the CS-nomogram was the individualized update of survival prognosis based on the time survivors survived since diagnosis. Previous cervical cancer nomograms only predicted a static survival rate and did not assess changes in survival after patients survived for several years (18, 19). This dynamic assessment approach might be more useful for follow-up monitoring and medical resource allocation.

CS analysis, calculated with the subset of survived patients, required much patient data support, so we chose the SEER database. Because data on survived patients diminished over time, and few medical centers could provide such a large sample. We have rigorously evaluated and validated the current CS-nomogram, which was quite stable and accurate in predicting patient CSS in real-time. The calibration curves that almost coincided with the ideal curve, the ROC curves that remained stable within 5-,10-, and 15 years and the DCA that showed high net benefits demonstrated its power. Thus, this novel model provided prognostic information consistent with real-time follow-up and had considerable clinical utility.

There were some limitations to our study. First, this retrospective study was inevitably biased. Secondly, the SEER database lacked some information [such as tumor markers, human papillomavirus (HPV) status, lymphovascular space invasion (LVSI) etc.], which may limit our analysis. Third, CS analysis required a large amount of data, so external validation was challenging. Fourth, updating this CS-nomogram over several years is necessary as treatment strategies improve.

## Conclusions

This study used CS analysis to explain the gradual improvement in CSS over time in long-term survived cervical cancer patients and found that when survival exceeded 5-6 years, the patient’s risk of death from cervical cancer would be less than 5% in 10-15 years. Meanwhile, we developed the first novel CS-nomogram for predicting survival in individualized and real-time. This model showed strong performance and provided more dynamic prognostic information with long-term surviving patients. In addition, this tool required external validation for its generalization.

## Data availability statement

The raw data supporting the conclusions of this article will be made available by the authors, without undue reservation.

## Ethics statement

Ethical review and approval was not required for the study on human participants in accordance with the local legislation and institutional requirements. Written informed consent for participation was not required for this study in accordance with the national legislation and the institutional requirements.

## Author contributions

XM and YG: designed the study. XM, XC, and YJ: performed the study and analysed the data. XM: wrote the manuscript. YJ and YG: provided the expert consultations and clinical suggestions. XM and YZ: conceived of the study, participated in its design, and coordination. XC, YJ, and YG: helped to draft the manuscript. All authors contributed to the article and approved the submitted version.
