# Supplementary material for: Conditional survival analysis and real-time prognosis prediction for cervical cancer patients below the age of 65 years
Source: Front Oncol. 2023 Jan 9;12:1049531. doi: 10.3389/fonc.2022.1049531 (PMC9868950; doi:10.3389/fonc.2022.1049531)
Supplement: Supplementary file 1 [file DataSheet_1.docx]

Supplementary Material

Conditional survival analysis and real-time prognosis prediction for cervical cancer patients below the age of 65 years

Xiangdi Meng^1^, Yingxiao Jiang^1^, Xiaolong Chang^1^, Yan Zhang^2^, Yinghua Guo^1^*

*** Correspondence:** Yinghua Guo, guoyinghua_wfph@163.com

## Supplementary Figures


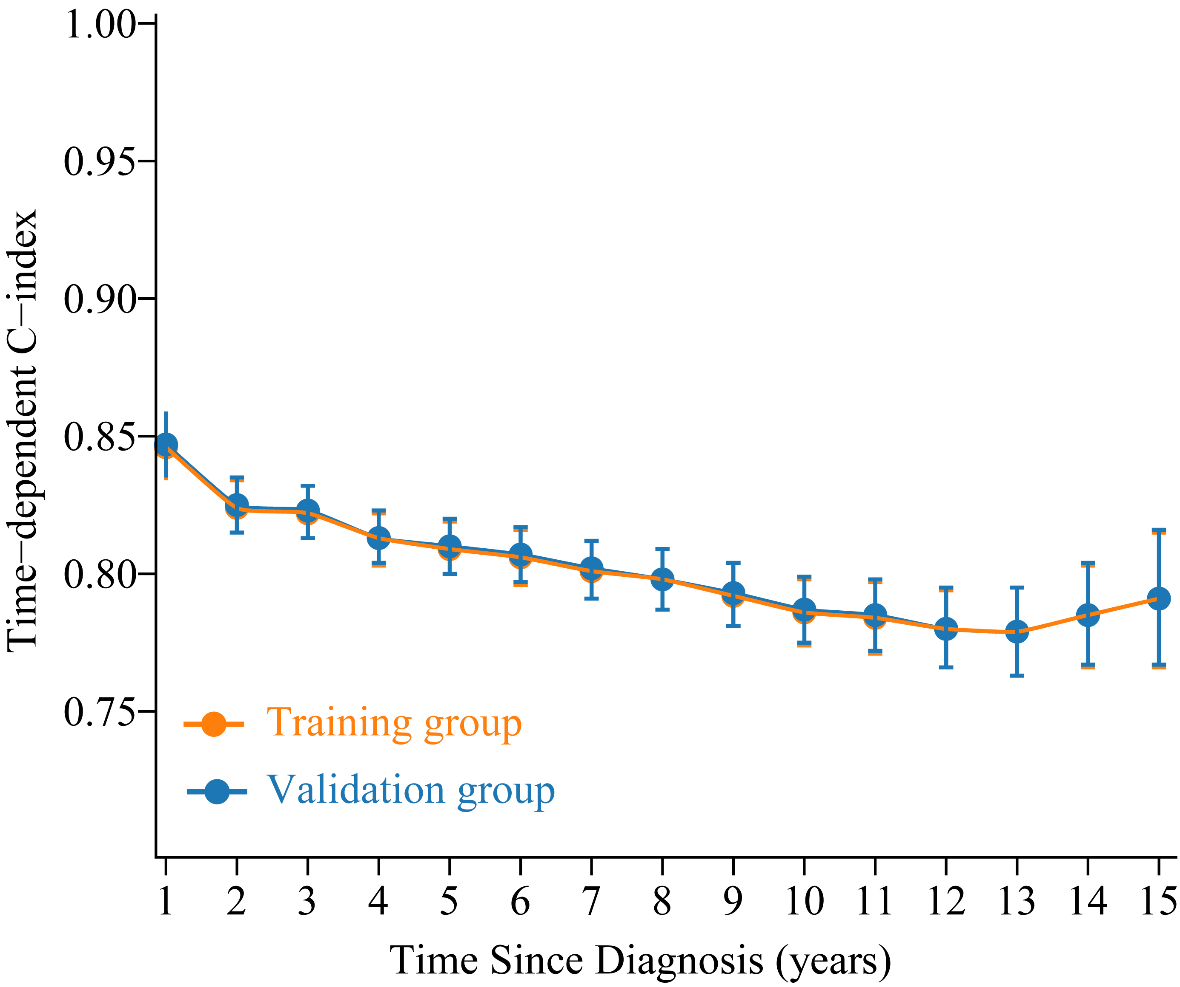


**Supplementary Figure 1.** Time-dependent C-index of the CS-nomogram
